# Treatment of Miller I Mandibular Gingival Recessions Using PRF vs. Connective Graft

**DOI:** 10.1155/2021/6616688

**Published:** 2021-04-08

**Authors:** Hernan S. Garzon, Camilo Alfonso, Francisco J. Vega, Andrea García, Ana Muñoz, Gustavo Jaimes, Katherine Isaza, Katherine Rivera

**Affiliations:** Grupo de Investigación en Salud Oral, Facultad de Odontología, Posgrado en Periodoncia, Universidad Antonio Nariño, Bogotá, Colombia

## Abstract

Gingival recession (GR) can cause aesthetic and functional problems. Using connective tissue graft (CTG) and coronally advanced flap (CAF) is considered the technique of choice for treating GR. Considering the morbidity resulting from taking CTG, different alternative biomaterials have been described, including plasma-rich fibrin (PRF) membrane. Studies in lower teeth are few because of the complexity of the factors that can influence obtaining less predictable outcomes. *Objective*. To compare between CAF + PRF and CAF + CTG in the treatment of lower teeth Miller I gingival recession. *Methodology*. Split-mouth included 26 isolated GR (13 in each side of the mouth). The left side was treated with CAF + PRF and the right side with CAF + CTG. Clinical variables, probing depth (PD), GR, keratinized tissue (KT), vestibular soft tissue thickness (VSTT), and teeth sensitivity (TS), were assessed at the baseline. GR, KT, VSTT, extraoral inflammation (EI), and patient discomfort (PaD) were assessed at 45 days. *Results*. Statistically greater VSTT at 45 days was obtained using CAF + CTG (*p* < 0.05). Less EI and PaD were obtained using CAF + PRF (*p* < 0.05). No change was observed in GR, KT, and TS values in the intergroup or intragroup comparisons. *Conclusion*. Even with the limitations of this study, using PRF and CTG in lower teeth demonstrated an improvement in terms of root coverage, although it was without a total percentage of coverage. Regarding the VSTT, better results were obtained using the CTG + CAF, suggesting eventually long-term stable clinical results. We suggest a combined technique for future investigations.

## 1. Introduction

Gingival recession (GR) is defined as the displacement of the gingival margin to the apical side of the cementoenamel junction (CEJ) [1]. It usually triggers predisposition to root caries, dental hypersensitivity, and inadequate control of dental biofilm formation and loss of periodontal attachment [2]. GR implies aesthetic and functional problems with evidence of progress over time [3]. Treatment of GR has become a research focus in periodontal plastic surgery as it is present in postorthodontic patients or those who anatomically challenging mucogingival conditions associated with a thin gingival phenotype or trauma of brushing [4]. Another condition associated with the presence of gingival recessions is periodontal disease or its sequelae. The most common presentation is interproximal attachment loss, but it is also vestibular or palatal. The role of inflammation in gingival recessions favors the growth and anastomosis of the rete pegs. Furthermore, this inflammation may favor the destruction of connective tissue and bone resorption mediated by proinflammatory cytokines through cellular activation. As a result of this chronic inflammatory process and even its therapy, it influences the appearance of gingival recessions [5, 6].

Possible predictors of several techniques for the treatment of recessions have been proposed.

The current technique of choice is CAF + CTG with a predictability of 73%–96% in root coverage and stability for 20 years [7–10]. On the other hand, the use of CTG has limitations such as postoperative pain, possibility of bleeding, volumetric changes in the palate, and discomfort for the patient [11, 12].

Due to the morbidity of the donor site, substitute biomaterials have been proposed such as dermal allografts (acellular dermal matrix) [13], with a radicular coverage rate like autologous grafting. However, systematic reviews show a difference in keratinized tissue gain and complete root coverage, being greater with the use of CTG [14, 15]. Collagen matrices of porcine origin have been used, showing 84% of root coverage at six months and 89% at one year in combination with a CAF. Perhaps, the CTG obtained greater results: 97% of radicular coverage at six months and 99% at one year [16, 17].

PRF has been described as an autologous membrane source of growth factors, associated with soft tissue regeneration processes, in hard tissues, and this needs more investigation [18]. It has biological modifiers such as platelets [19], IL-4, IL- 6, IL-8, IL-10 [20], stem cells, leukocytes embedded within a fibrin matrix, PDGF, TGF-*β*1, IGF, and VEGF [21]. The regenerative potential of these cytokines has been studied in wound healing. PRF could release growth factors and glycoproteins for approximately 7–10 days after surgery. PRF influenced the regulation of immune response, and in the remodeling of the matrix during the healing and synthesis of vascular tissue, it reduces inflammation and postsurgical discomfort [22].

PRF has been used for the treatment of gingival recessions. A percentage of root coverage is evident from 75% to 91% [23]. Its use can be an alternative to CTG, with even similar results [24, 25]. The literature reports variability in the results with the use of PRF for root coverage. However, many authors confirm the possible benefits of using PRF + CAF [26].

However, the use of these biomaterials in lower teeth gingival recessions requires additional research, due to the challenges that this area presents. Some conditions influence the success of the treatment probably due to the poor mucogingival conditions of the area, and this includes the presence of deep recessions [27]. Moreover, the scarce depth of the vestibule and shallow quantity of keratinized tissue cause a coronal muscular insertion. This “marginal muscular insertion” is considered a difficulty when getting the free muscle flap and trying to retard the reattachment [28]. The rationale of this study is that there is scarce evidence for the management of gingival recessions in the lower teeth using a substitute material for the CTG, such as PRF. The aim of the study was to compare the CAF + PRF and CAF + CTG for the treatment of lower teeth Miller I gingival recession. The study included patients affected by bilateral Miller I gingival recessions in the lower teeth. The null hypothesis is that the use of the CTG does not present advantages over the use of PRF to treat gingival recessions in the clinical variables measured in the study.

## 2. Materials and Methods

Seven subjects, three males and four females, affected by bilateral Miller I gingival recessions in the lower incisors or canines were included in the study. The patients attended the Periodontics Clinic at Antonio Nariño University, Bogotá Colombia, in the period between June 2017 and June 2018. The selected patients gave their written informed consent in accordance with the Declaration of Helsinki. The study protocol was approved by the Ethics Committee of the Antonio Nariño University, Colombia (code: 1509-2017).

All the participants had to meet the following inclusion criteria:

- Being over 18 years of age
- Systemically and periodontally healthy
- Plaque index (PI) less than 15% (Silness & Löe)
- Presence of isolated bilateral Miller I gingival recessions at the buccal aspect and an identifiable CEJ of lower incisors and canines
- Nonsmokers

### 2.1. Study Exclusion Criteria

  The exclusion criteria were as follows:

- Pregnancy or breastfeeding
- Consumption of antiplatelet drugs or anticoagulants
- Using antiepileptic drugs
- Using antibiotics for at least 20 days before surgery

### 2.2. Experimental Design

The study was a blind, prospective, split-mouth, controlled pilot clinical trial, comparing the use of CTG and PRF for the treatment of lower teeth gingival recessions. Each patient had two or more gingival recessions (contralateral). On the left side, PRF was used, and on the right side, CTG was used. Period of observation was 45 days.

According to the protocol of the study, the following phases were performed.

#### 2.2.1. Initial Therapy and Clinical Measurements

All patients received oral hygiene instructions, scaling, and root planning 20 days before the procedure and had a modification of habits related to the etiology of gingival recessions (traumatic tooth brushing or flossing). Surgical treatment of the recession defects was not scheduled until the patient could demonstrate an adequate standard of supragingival plaque control.

#### 2.2.2. Investigator Training

All participating investigators were required to attend three training and calibration meetings. The aims of the meeting were to review the protocol and standardize the case selection.

All pre- and postsurgery measurements were carried out by a single calibrated blind examiner (E1). He did not perform any surgery or intervention on the patients. Measurements of GR, PD, and KT were repeated three times by the examiner for a total of 50 defects in the patients of Dental Clinic at Antonio Nariño University with a Kappa of 0.82.

#### 2.2.3. Clinical Variables

Clinical variables, PD, GR, KT, VSTT, and teeth sensitivity (TS), were assessed at the baseline. GR, KT, VSTT, extraoral inflammation (EI), and patient's discomfort (PaD) were assessed at 45 days after surgery.

The PD, GR, KT values were performed at the midbuccal aspect of the treated teeth with a North Caroline probe graduated in increments of 1 mm and rounded to the nearest millimeter (Hu Friedy, PCUNC15 USA).

VSTT was measured with a # 40 endodontic file across the gingival tissue perpendicularly 2 mm below the gingival margin under local anesthesia, and then, the thickness was measured using a digital caliper (Truper).

The variable TS was evaluated by a binary scale: presence or absence of dental sensitivity to cold or heat (0 = no; 1 = yes).

EI and PaD were measured by comparing visual analog scale scores.

### 2.3. Surgical Procedure

#### 2.3.1. PRF Collection

**Figure 1:**
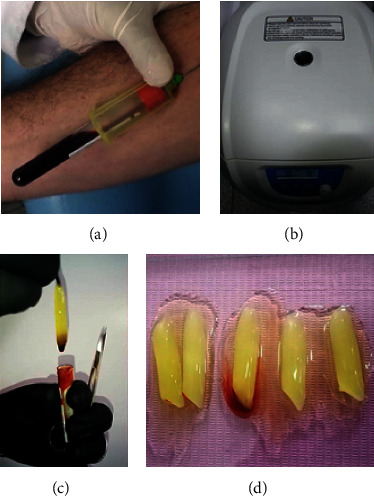
PRF collection. A blood sample was obtained in 10 ml plastic tubes without anticoagulant and no activator. Immediately, the tubes were centrifugated at 3000 rpm for 10 minutes (Scilogex DM1536). The fibrin clot was removed from the tube and separated by microsurgical scissors. The PRF membrane was pressed into a calibrated metal box that forms membranes at a constant thickness of 1 mm. Two membranes of PRF were superimposed and immediately taken into the surgical site.

For the collection of PRF membranes, Choukroun's protocol was used [29] (Figure 1).

#### 2.3.2. CTG Collection

CTG was obtained from the palate using the envelope technique described by Langer and Langer [30].

#### 2.3.3. Surgical Technique

All surgeries were performed by the same expert periodontist (HG).

The surgical technique adopted in the test defects was the trapezoidal type of CAF (De Sanctis and Zuchelly, 2007) fully covering a CTG or PRF. The labial submucosa tissue was removed, according to the technique of Zuchelli et al. [28].

Mechanical treatment of root surfaces was performed and EDTA 24% for two minutes was used after surgical exposure of the root.

On the left side, two PRF membranes were used and on the right side, CTG (2 mm thickness); both techniques included a CAF. CTG or PRF was fixed to the interdental papillae and adjacent soft tissue in the apical portion with absorbable 5-0 suture and using horizontal mattress technique. The flap is repositioned coronally to the cementoenamel junction and was sutured using 5-0 polypropylene with suspensory technique.

#### 2.3.4. Postoperative Care

Brushing in the area should be avoided until complete removal of the suture (6 weeks). Ice should be applied to the surgical area in the face during the first 48 hours. A soft diet should be consumed during the first week. Ibuprofen 600 mg should be administered, and the patient should rinse with chlorhexidine (0.12%) three times a day for one minute. Postoperative follow-up was at the seventh and fourteenth days. This protocol was the same for all patients.

#### 2.3.5. Statistical Analysis

The SPSS V22 program was used. Shapiro–Wilk tests were developed to verify normality in the data. After the normality assessment using the Shapiro–Wilk test, the data were analyzed with the Mann–Whitney U and Kruskal–Wallis ANOVA tests to assess the intergroup differences. Wilcoxon test was used to find intragroup differences.

## 3. Results

No case was lost during the study. No postoperative complications were presented. All patients had <10% plaque and bleeding on probing before the surgery.

Descriptive data for clinical parameters, PD, GR, KT, VSTT, TS, EI, and PaD, are summarized in Table 1.

The ANOVA test of Kruskal–Wallis determined (significance of *α* = 0.05) that, regarding the postoperative GR at 45 days, there was no significant statistical difference between the inter- and intragroup with the use of CTG or PRF. However, complete root coverage was not assessed in any group (CTG = 87.9%; PRF = 84.6%).

Mann–Whitney *U* test was performed to compare changes in VSTT, EI, and PaD. We found a significant statistical (*p* < 0.05) difference between the CTG and PRF from baseline to 45 days. A greater VSTT was found in the CTG group at 45 days after surgery. There were less EI and PaD with the use of CAF + PRF (*p* < 0.05).

## 4. Discussion

In this study, the variable which was statistically different between groups was a greater VSTT using CTG. The clinical implications and advantages of PRF are related to avoiding a donor site, release of growth factors during the initial postsurgery days, being a matrix to the stabilization of a clot, and reducing the inflammation [12]. According to that, we expected quite less healing time in the PRF group or a fast degree of keratinization, but, in this short period of time, these characteristics were no different. Less EI and PaD using PRF were expected, and this was consistent with other [31]. These could be explained by the fact that PRF release immune-modulatory cytokines, and the thickness consisted of two layers, which was thin.

The treatment of gingival recessions in the mandible involves some anatomical conditions that imply a more complex approach to achieve a successful result. Most of the literature about the treatment of gingival recessions include upper teeth. The results of this study showed that CTG or PRF is effective for the treatment of Miller I recessions when the labial submucosa tissue was removed [28] even in the two groups with *α* <2 mm of KT although this was without a total percentage of coverage. This result is consistent with that of some authors, who confirm that the use of any of the two autologous biomaterials does not present differences in the percentage of root coverage in upper gingival recessions [10, 13]. Because of the limited time of observation (45 days), the final KT was not evaluated, but it was expected to be more in the CTG group [16, 17].

However, statistically significant differences were found with respect to the soft vestibular tissue thickness at 45 days, where the CTG + CAF technique presented better results. This soft tissue vestibular volume could be a predictor of future gingival recession or probably of relapse.

GR has to do with deficiencies in the alveolar bone, either anatomical or acquired. It can exist in the absence of periodontal disease or occur as a sequel to it [1], during which there is loss of periodontal tissue, and the release of cytokines such as IL–1, IL–2, IL–17, TNF-alpha, and PGE2 could promote hipoxia, oxidative stress, relative vasospasm, increased coagulation, and endothelial injury [5, 32]. Therefore, it is important to generate mechanisms for early detection of periodontal disease, using salivary or blood markers that allow early treatment and prevent the progression of the disease that could influence the GR prevalence [33].

The need for a meticulous evaluation of the periodontal phenotype before any intervention in the treatment of lower teeth recessions is obligatory. In a thin gingival phenotype, the technique of choice will probably be the Gold Standard. Although the use of PRF would not be contraindicated, the possibility of recurrence of recession would be greater in the future because of the final thin VSTT. The periodontal phenotype is a predictor of the result [34]. Moreover, it is necessary to consider the keratinized tissue before any surgery [35].

The thickness of the PRF membranes was one of the variables which has not been considered in this study and requires further investigation. The current evidence in controlled clinical trials shows that using a greater number of layers gives a better result [36]. In a thin gingival phenotype, the use of exaggerated layers of PRF could predispose to excessive tension in the flap and the failure of the surgery. We found that, to treat gingival recession, the use of two layers is suitable and that, in order to improve the gingival phenotype, the decision could change to using CTG.

Finally, we suggest a combined technique because, nowadays, CTG is not necessary to obtain more than 1 mm of thickness for successful results with site-specific application [37]. This implies less pain to the patient and controlled bleeding. Moreover, if it is possible, PRF membrane or injectable PRF can be used in the donor and receiving site in order to take advantage of the regulation cytokines and healing components that allow less postoperative pain and discomfort [38]. Those autografts are not antagonists; the combination could have better results and need more investigation. In addition, an evaluation of the creeping attachment in the patients of this study is suggested.

## 5. Conclusion

Different alternative biomaterials have been described, including the PRF membrane in periodontal plastic surgery as an alternative treatment to reduce morbidity and complications caused by taking a connective tissue graft. Studies in lower teeth are few due to the complexity of local factors that can influence obtaining less predictable results.

Although there were limitations in this study, including short observation time in the initial healing process, using PRF and CTG in lower teeth demonstrated an improvement in terms of root coverage although it was without a total percentage of coverage. Regarding the vestibular soft tissue thickness, better results were achieved using the CTG + CAF, which could eventually mean obtaining long-term stable clinical results and a less probability of future recession. We suggest a combined technique for future investigations.

## Figures and Tables

**Table 1:** Descriptive data for clinical parameters.

	Connective tissue graft group (*n* = 13 cases)	PRF membrane group (*n* = 13 cases)
PD (probing depth) (mm)		
Baseline	1.23 ± 0.44	1.38 ± 0.65
Recession depth (RD)		
Baseline	3.15 ± 1.40	3.00 ± 1.58
45 days	0.38 ± 0.50	0.38 ± 0.51
Difference	2.77 ± 0.89	2.54 ± 1.06
Keratinized tissue (KT)		
Baseline	1.27 ± 0.44	1.12 ± 0.22
Vestibular soft tissue thickness (VSTT)		
Baseline	1.17 ± 0.30	1.19 ± 0.30
45 days	3.67 ± 1.29	1.26 ± 0.28
Difference	−2.49 ± 0.99*∗*	−0.07 ± 0.02*∗*
Tooth sensitivity (TS)		
Baseline	100% no	100% no
7 days	100% no	100% no
45 days	100% no	100% no

*∗*Between-group statistical difference.

## Data Availability

The data used to support the findings of this study are available from the corresponding author upon request.
